# Round the Hospitals

**Published:** 1920-05-15

**Authors:** 


### ROUND THE HOSPITALS.

Two military Investitures were held last week by Prince Henry, officiating for Prince Arthur of Connaught. The first was on May 6, at Aberdeen, where the Marquis of Aberdeen and Lieut.-General Sir Francis J. Davies received the young Prince and escorted him to the town and county hall, where a large company assembled to witness the ceremony. There were 101 persons to receive decorations, and among them were ten ladies, three of whom received the Order of the British Empire and the remaining seven nursing sisters that of the Royal Red Cross. Prince Henry held a larger Investiture the following day at Newcastle-on-Tyne, when 200 persons received decorations at his hands in the King's Hall of the Armstrong College. The students turned out in great numbers to accord the Prince such tokens of loyalty as accorded with their high spirits, and the occasion was in all respects highly interesting and cheerful.

**ROUND THE HOSPITALS**—(*continued*).

The Investitures, to be resumed this week, will for the future take place, weather permitting, in the open air, and this decision of the King has given great pleasure, for it enables outside members of the public to witness some part, at any rate, of the ceremony. Those who are to receive decorations have the privilege of special invitations for relatives and friends, who are accommodated with seats in the Palace Quadrangle. Outside the grand entrance of the Palace a platform with a canopy has been erected for the bestowal by the King of the various orders and decorations, and below, in the Quadrangle, the military band will play.

Princess Mary donned her V.A.D. uniform on May 8 and visited Mornington Crescent to take part in the demonstrations carried out in connection with St. Pancras Health Week. It was anything but royal weather. Nevertheless, large crowds assembled in the rain to cheer the Princess, and neither the mothers nor the babies were to be deterred from meeting their royal visitor. The exhibit, which showed one of the invalid kitchens of London at work, specially engaged the attention of Princess Mary, and she charmed the demonstrators by her close and practical interest in the dishes under preparation. Her Royal Highness stayed some time among the mothers, whose little ones were in the big marquee, and gave great pleasure by the friendly simplicity of her talk. Before leaving the Princess was present at parades of the Boy Scouts, the Girl Guides, and the aeroplane construction workers.

The centenary of Florence Nightingale's birth, which is being commemorated as we go to press at Westminster Abbey (see page 170), is the occasion for an earnest appeal for the Nation's Tribute to Nurses, backed by the influential names of Dame Ethel Becher, Matron-in-Chief, Q.A.I.M.N.S. (retired); Dame Sidney Browne, Matron-in-Chief, T.F.N.S. (retired); Dame Maud McCarthy, Matron-in-Chief, B.E.F. (retired); Dame Sarah Swift, Matron-in-Chief, Joint War Committee; Miss Lloyd Still, Head of Nightingale School, and Miss C. May Beeman, Hon. Organiser of Nurses' Tribute. The entire scheme of aid projected on behalf of nurses can be realised with one million shillings (£50,000), and, owing to a generous donation to the College of Nursing, only 400,000 more shillings are required to close the fund. Strenuous efforts, in which the Hon. Sir Arthur Stanley is taking a part, are being made this week for this purpose, and all who can help, to however small a degree, should do so. In days to come they will be proud to think that they lent a hand to this great endeavour on behalf of the nurses whose sufferings were incurred in alleviating the horrors of war. The querulous murmurs of opposition to this just and necessary effort have died, half ashamed, away. The total amount acknowledged to date by the *Daily Telegraph* is 241,714 shillings.

No one can read the fifth annual report of the Council of the College of Nursing, published on page 183, without a feeling of intense satisfaction at the amount of good work which has been accomplished during the past year. It is a record of progress all along the line. At the close of the financial year (March 31, 1920) 17,336 general-trained nurses had enrolled as members, no fewer than 4,322 having joined during the year. We defer detailed consideration of the reports and accounts, and, meanwhile, congratulate the members of the College of Nursing on the solidarity which has been exhibited within the College and on the increased activity and interest in the conduct of their own affairs displayed by individual members. The fifth ordinary general meeting of the College, at which the annual report and balance sheet will be submitted for adoption, will be held on Thursday, June 17, at 3 p.m., at the Royal Society of Medicine, 1 Wimpole Street, W. 1. The Conference to be held the same evening at 7.30 p.m. and on the following day at 11 a.m. will include discussions on subjects of considerable interest to the nursing world. At the annual general meeting the result of the voting for the election to the Council of twelve members will be made known. Fifty candidates have been nominated in the English and Welsh section, ten in the Scottish section, and six in the Irish section. The majority of candidates nominated are matrons, but the list includes a nurse, three Queen's nurses, a health worker, a school nurse, and a tuberculosis health visitor. The voting-papers have been posted to members, and must be received back by the Secretary of the College at 7 Henrietta Street, London, W. 1, not later than Monday, May 24; or, if from abroad, by Tuesday, June 1.

The College of Nursing makes important announcements this week of two studentships for the training of health visitors under the regulations of the Board of Education and for the course for sister-tutor. Candidates for the health visitor studentship must be trained nurses, members of the College of Nursing, and holders of the certificate of the Central Midwives Board. The course, taken at the Household and Social Science Department, King's College for Women, Campden Hill Road (or such other centre in Scotland or Ireland as may be arranged), is for one year, and the value of the studentship is 112 guineas, which includes the payment of college fees and board and lodging. A certificate will be awarded by the Board of Education to those students passing the required examinations. Applications for studentships must be received by the Secretary of the College of Nursing by June 11, and a qualifying examination will be held on June 26 in England, Scotland, and Ireland respectively. The total number of marks gained in the qualifying examination is only one factor in the selection of the successful candidate, whose past experience and references are also taken into consideration.

**ROUND THE HOSPITALS—(*continued*).**

In addition to sister-tutor studentships to be awarded by the College of Nursing, there are two Cowdray studentships. Each is of the value of 112 guineas, which includes the payment of college fees and board and lodging. The course is for one year, and is held at the Household and Social Science Department, King's College for Women, Campden Hill Road. A certificate will be awarded by King's College to those students who pass an examination at the end of the session. The session, as in the case of the health visitor course, opens in October. Candidates must be trained nurses, members of the College of Nursing, and priority will be given to those having experience in sister's duties. The Cowdray studentships will primarily be reserved for those members of the College of Nursing who have received their professional training in Aberdeenshire or Sussex. Applications for either of the studentships must be received by June 11, and a qualifying examination will be held on June 26 in England, Scotland, and Ireland respectively. The same discrimination on the part of the Selection Committee applies to the candidates for sister-tutor studentships as to those for the health visitor studentships. Any trained nurse wishing to qualify as a sister-tutor independently of the above studentships is admitted to the course on payment of the fees. Application should be made in such cases to the Dean of King's College, Campden Hill Road, London, W.

There does not appear to be any very great demand for midwifery scholarships. Recently the Stretford Education Committee recommended the Lancashire County Education Committee to consider the desirability of increasing the number of such scholarships to eight. The Lancashire Committee, however, has considered the point, but, in view of the small number of entrants for this class of scholarship during past years, concludes that there is no need to increase the number.

The promoters of the proposed V.A.D. Club are calling a halt, prior to securing the necessary capital for establishing it on a sound financial basis. To secure a lease for 999 years of the selected hotel in Cavendish Square, with its furniture, the sum of £25,000 is required. Of this only £6,000 is forthcoming, and nothing can be done until at least another £10,000 is paid into the funds. The club invites subscribers for 15,000 £2 shares, and we cannot but believe that there are more than double that number of persons willing to lend their support to such a useful project. Unfortunately, the option on the Cavendish Square premises expired at the end of April, so prospects of an immediate opening are not promising.

The conference held on Thursday, Friday, and Saturday this week at the Albert Hall, Manchester, "On the Prevention of Diseases of the Teeth" is promoted by Mr. Charles E. Hecht, of the Food Education Society, and should prove attractive to a large circle of health workers. Nurses in uniform and health visitors are admitted free. Popular papers are to be read, round which discussion will centre. There is an exhibition and a bookstall, where useful books and pamphlets may be examined and acquired. Manchester is a very good place to select for this useful gathering, for its people have a reputation for the open ear and the seeing eye. It is high time that food reformers should concentrate attention on the question of teeth preservation, for the best authorities are united in believing it to turn on sound principles of nutrition, just as nutrition itself depends largely on the possession and right use of good teeth.

We regret to record the sudden death of Miss Bessie Carley, R.R.C., aged thirty-eight, lately appointed matron to the newly opened Streatham Red Cross Medical and Surgical Home. She was seized with illness on May 2 and removed to Guy's Hospital, where she died the following day. Miss Carley was a member of the Territorial Force Nursing Service, and served in France for two years as assistant matron. She held the C.M.B. certificate and was a skilled masseuse. Her loss is deeply felt at the Home, where she had many friends and was greatly valued.

Universal satisfaction will be caused by the announcement we are able to make this week that Miss Eleanor C. Barton, Matron of Chelsea Infirmary, has been induced to withdraw her resignation and will continue to hold the office she has been filling with such conspicuous success. We trust that arrangements have been made to ensure her an adequate holiday and complete rest with increased assistance when she resumes her duties. Hospital work, and in particular the direction of the nurse training-school, make much greater demands on the superintendent than they used to do, and the Chelsea Guardians, who have always moved with the times, no doubt apprehend the fact that there are limits to the expansion of human energies. It is a great achievement that they have secured an extension of Miss Barton's valuable services for their institution.

Sir Edward Stern, the City financier, has given £5,000 Consols to the Chertsey Nursing Association, the interest on which is to provide the entire cost of an additional nurse. The donation is given in memory of his late wife, who was President of the Chertsey Nursing Association. Sir Edward Stern is a firm believer in voluntary hospitals and nursing associations, and opposed to their absorption by the State. "Depend upon one thing," he said recently; "work done voluntarily is more kindly and unselfish than work done by the State. State work is more expensive, and infinitely more ruthless."

The three days May fair and sale, held last week in the Parochial Hall of St. John the Divine, Kennington, for the benefit of the Belgrave Hospital for Children, was exceedingly well got up, and proved

**ROUND THE HOSPITALS**—(*continued*).

a great attraction. The stalls represented an old English village and no telling detail was omitted. The victim in the stocks guarded by the beadle was particularly realistic and brought much grist to the mill. Katherine Duchess of Westminster was unfortunately prevented by illness from opening the fair on the first day, and her place was taken by the Countess of Leicester.

A former music-hall singer, staying at the Y.M.C.A. at Victoria, has been fined £20 for parading the streets in the hospital blue selling post-cards. It appeared that he was only 25 days in France when he is said to have been buried by a bursting shell as he was leaving his dug-out. He was subsequently discharged with a pension of £2 a week. Since then he has done no work, but he did pretty well by selling the post-cards, for he had £48 when arrested, and it was shown that he had been changing about £4 worth of coppers daily. The cards he sold gave his military record, but omitted to mention the trifling fact of his pension. There is something peculiarly offensive in such a profanation of the familiar almost sacred blue.

Sir Arthur May, who presided at the annual meeting of the Cornwall County Nursing Association, spoke in a tone of great depression and discouragement. He reported that not only is there a great dearth of Cornish women desirous of being trained for work in the county, but that the spirit of service is very low right through the community. Only nine probationers were trained last year, and if there had been more applicants for training there was no money wherewith to carry out the training. Even the secretaries of affiliated associations were apathetic and indifferent, if not actually antagonistic, to health visiting and school nursing. Very little progress has been made in regard to starting work in areas hitherto unnursed, but six new district associations have been formed, and there are now 108 in the county. Owing to the small number of candidates sent in for training there will be a shortage of village nurses throughout the year. The British Red Cross Society has made a grant of £3,000 which has been invested, the Local Government grant is £120 more than before, and the County Council grant £400 more. Frankly, we do not believe Cornish folk are less accessible to the voice of humanity than the inhabitants of other parts of England. We are tempted to suspect the lack of initiative and energy at headquarters and the absence of any imaginative quality in the appeals either for money or nurses.

The new Municipal Maternity Hospital at Portsmouth, the fulfilment of a scheme long deferred owing to the war, was opened on April 19 by the Mayor of Portsmouth, Councillor Timpson, accompanied by the Mayoress. The Ravenscourt Hospital contains three wards, with twelve beds in all. There are well-appointed labour and isolation wards, good rooms for the matron and the two nursing sisters, and convenient administrative offices. The five probationers are housed in cubicles, not a very satisfactory arrangement, in a large hut erected in the garden, but take their meals in the main building. We fear it may be difficult to prevent those on night duty from disturbing the others under these conditions. Patients are to pay 30s. a fortnight. The rate of infant mortality in the borough is still 100 per 1,000, reduced of late years from 150 per 1,000, and the rate of mortality among mothers is far higher than it ought to be, very largely owing to defective housing. Portsmouth has already a Maternity Hospital, opened in 1907, and under the control of the Board of Guardians. There is abundant evidence that the new institution is greatly needed for the accommodation of mothers able to pay a considerable part of the cost yet whose homes are crowded and insanitary.

Canadians are much more whole-hearted in their schemes for Red Cross work than the English. The Canadian Red Cross Society has set itself the task of enlisting the entire population in its membership. The idea is less to collect funds than to create sympathy and secure moral support. The means adopted are to be the familiar "drive," and a week has been set apart—October 4 to 11—for the prosecution of a general campaign throughout the Dominion. The aim is a healthy Canada, a Canada which will lead the world in its low percentage of preventable disease. The promotors of the movement rely largely on the junior organisation for attaining their ideal. This branch of Red Cross activity has been strangely neglected in this country, and we might well take a lesson from Canada and strive to enlist the sympathies and services of our young people on behalf of suffering children. We are strongly of opinion, however, that children should not be required or encouraged to collect money.

The Surrey Red Cross Society has issued its report for 1918-19. It testifies in an uncommon degree to the public spirit of the county in the period included. At the time of the armistice there were fifty-three hospitals in Surrey under the jurisdiction of the County Director, and by January in this year these had been reduced to a ward of thirty beds in the large hospital at Brooklands. The grand total of patients treated in 1915-1918 was 60,524. During the "Surrey Week," when a rally was made to secure funds, the sum of £40,438 was raised. Curative posts for providing electrical and massage treatment for ex-Service men have been opened at Redhill, Dorking, Farnham, Kingston, Woking, and Epsom, and another is in formation at Weybridge. Moreover, certain beds are being maintained in the County Hospital at Guildford for the use of ex-Service men, their wives and families, and this is combined with a scheme for giving training in the hospital to V.A.D. members. The leaders of the movement appear to be still as live as in war time, and this is much. Brigadier-General Scudamore has been appointed the new County Director.

